# A Review on Development of Industrial Solid Waste in Tunnel Grouting Materials: Feasibility, Performance, and Prospects

**DOI:** 10.3390/ma16216848

**Published:** 2023-10-25

**Authors:** Bolin Jiang, Mengjun Wu, Shanshan Wu, Aichen Zheng, Shiyong He

**Affiliations:** 1 Chongqing Vocational Institute of Engineering, Chongqing 402260, China; bolinjiang@cqvie.edu.cn (B.J.); aichenzheng@cqvie.edu.cn (A.Z.); 2 China Merchants Chongqing Communications Technology Research & Design Institute Co., Ltd., Chongqing 400067, China; mengjuwu@163.com; 3 School of Civil Engineering, Chongqing Jiaotong University, Chongqing 400074, China; shanshanwu84@163.com; 4 Chongqing Vocational College of Public Transportation, Chongqing 402247, China

**Keywords:** literature review, industrial solid waste, tunnel grouting materials, performance, applications

## Abstract

With rapid infrastructure development worldwide, the generation of industrial solid waste (ISW) has substantially increased, causing resource wastage and environmental pollution. Meanwhile, tunnel engineering requires large quantities of grouting material for ground treatment and consolidation. Using ISW as a component in tunnel grouts provides a sustainable solution to both issues. This paper presented a comprehensive review of the recent advancements in tunnel grouting materials using ISW, focusing on their feasibility, mechanical characteristics, and future development directions. Initially, the concept and classification of ISW were introduced, examining its feasibility and advantages as grouting materials in tunnels. Subsequently, various performances of ISW in tunnel grouting materials were summarized to explore the factors influencing mechanical strength, fluidity, durability, and microstructure characteristics. Simultaneously, this review analyzed current research trends and outlines future development directions. Major challenges, including quality assurance, environmental risks, and lack of standardized specifications, are discussed. Future research directions, including multifunctional grouts, integrated waste utilization, and advanced characterization techniques, are suggested to further advance this field. These findings provided useful insights for the continued development of high-performance and environmentally friendly ISW-based grouting materials.

## 1. Introduction

**Figure 1:**
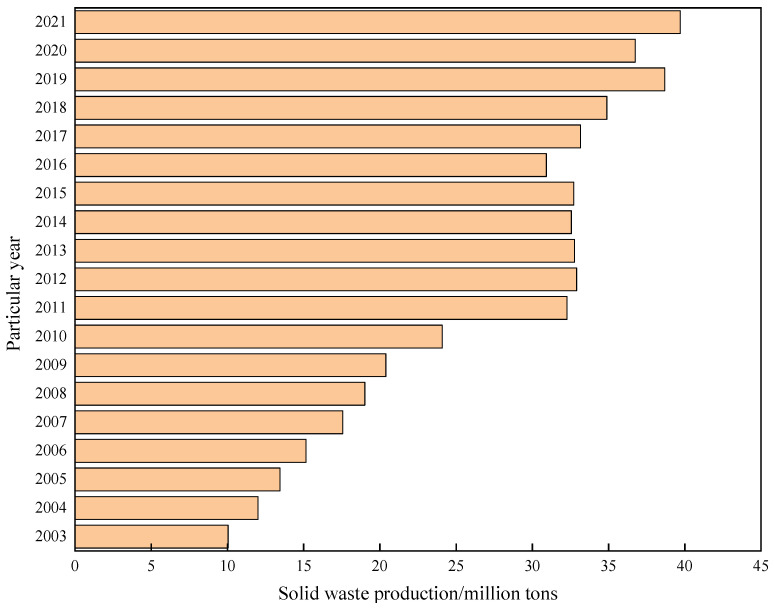
Annual output of solid waste in China.

**Figure 2:**
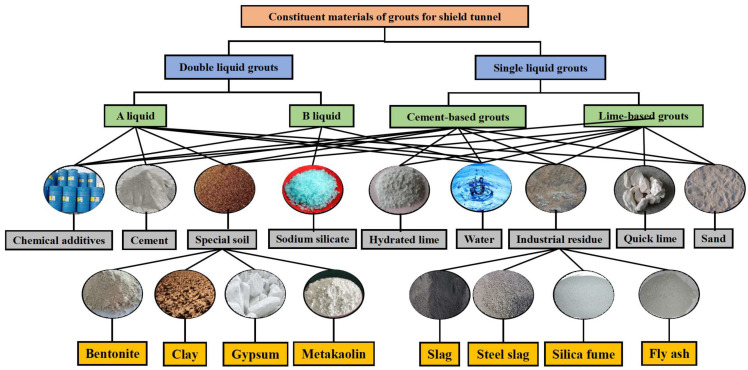
Composition of grouting material.

In recent years, infrastructure investment around the world has accelerated, resulting in a surge of tunnel construction projects for transportation, hydropower, and urban development [1,2,3,4]. Tunneling often takes place in soft ground or fractured rock, requiring large quantities of grouting materials for ground improvement and water inflow control [5,6,7,8]. Traditional cement and sodium silicate-based grouts used in tunnels incur high costs and substantial carbon emissions. Meanwhile, rapid industrialization and urbanization have led to a massive generation of industrial solid waste (ISW), including slag, fly ash, steel slag, red mud, etc. [9,10,11,12]. Annual global ISW production continues to increase, as shown in Figure 1. The annual production of ISW worldwide continues to increase, and China produces more than 10 billion tons of ISW annually [13,14,15]. Stockpiling these ISWs wastes resources and poses contamination risks to soil and groundwater. The requirement of reasonable disposal and the recycling of ISW is urgent in the world. Some studies reveal that ISW has the potential and feasibility to be used as grouting material (double-liquid grouts and single-liquid grouts) in tunnel engineering [16,17,18,19,20,21]. Therefore, increasing research efforts have focused on recycling ISW as substitute constituents in cementitious grouting materials; the details are shown in Figure 2. This emerging approach provides a promising solution to cut tunnel costs while reducing environmental pollution from ISW.

**Figure 3:**
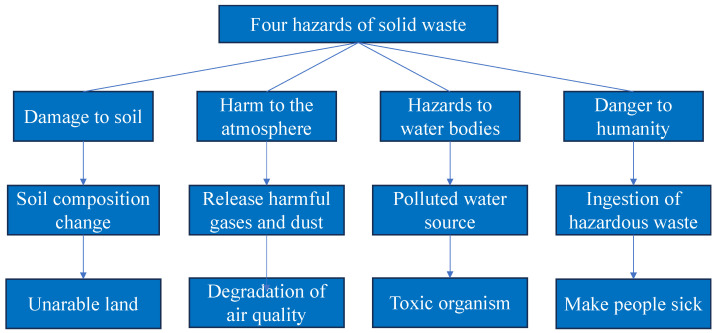
The four hazards of solid waste.

Compared to traditional grouting materials, ISW grouts exhibit certain advantages, such as high early strength, good impermeability, and improved durability [22,23,24,25]. Specific project examples are shown in Table 1. The complex chemical and mineral components in ISW can participate in pozzolanic reactions and generate cementitious gels. When rationally designed, ISW grouts can achieve equivalent or superior performance to conventional grouts [26]. However, utilizing ISW in grouting materials also faces some technical challenges. ISW comprises waste and by-products generated during industrial production processes, which contain various chemical components, resulting in different physical characteristics; the details are shown in Table 2. Due to the variability in ISW properties, obtaining a consistent grout quality that meets engineering requirements is difficult to guarantee; the details are shown in Figure 3 [27,28,29]. Furthermore, standardized codes and specifications for ISW grout production and application are still lacking. Zhang et al. [30] conducted a review study to examine the research progress of ISW as cementitious materials, highlighting various utilization methods and their application effects. As technology advanced and environmental concerns grew, research on ISW as grouting material gradually increased, leading to its practical application in engineering projects.

**Figure 4:**
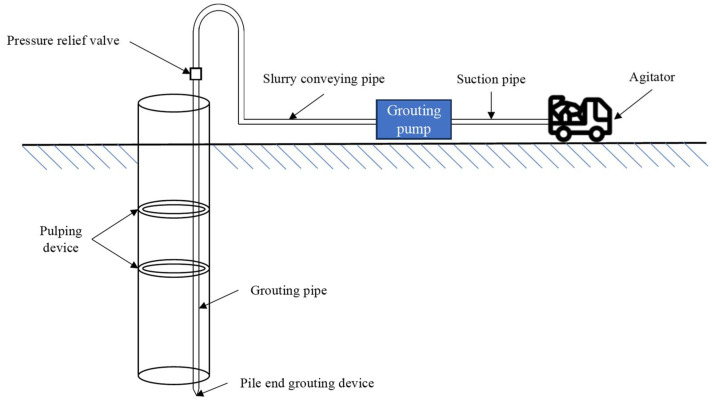
Schematic diagram of grouting process.

The potential and suitability of different ISW types in the production of grouting materials were explored by investigating their physical, chemical, and mechanical properties. For instance, waste slag, fly ash, and steel slag have been extensively studied and applied in grouting material preparation; the details are shown in Figure 4 [42,43,44,45,46,47]. Furthermore, the development of chemical grouting technology has opened up more possibilities for ISW utilization. Sun et al. [48] investigated the use of chemical grouting technology to convert waste steel slag into high-performance grouting materials. By adjusting the chemical formula and process parameters, they successfully transformed waste steel slag into grouting materials with exceptional properties; a range of 20–50% ISW content is proposed, which seems to be optimal for balancing the strength gain with the loss of workability and demonstrating the application potential of ISW in the field of grouting materials. Therefore, the research on utilizing ISW as a tunnel grouting material has undergone a significant and meaningful development process.

There is an increasing focus on the impact of ISW on the mechanical properties of grouting materials, prompting researchers to delve deeper by experimental and microscopic methods. Wang et al. [49] conducted experiments and simulation studies to investigate the influence of various waste components on grouting material performance, providing valuable insights for engineering applications. Ren et al. [50] examined the effects of different ISW components on the mechanical properties of grouting materials. They selected diverse ISWs, including waste slag, fly ash, and steel slag, and mixed them with cement to prepare a series of grouting materials. In a separate study, Sha et al. [51] focused on the influence of steel slag particle-size distribution on the mechanical properties of grouting materials. They prepared a range of grouting materials by sieving and classifying steel slag with varying particle size distributions. Additionally, Mirza et al. [52] investigated the effect of curing conditions on the mechanical properties of fly ash-containing grouting materials. They examined the impact of curing conditions on the strength, toughness, and deformation properties of these materials through compression and tensile tests. Therefore, a better understanding of the feasibility and optimization methods for utilizing ISW as a tunnel grouting material can be achieved, by studying the effects of different waste compositions, particle size distributions, and curing conditions on the mechanical properties of grouting materials.

The authors of this study concentrated on publications containing keywords such as “industrial solid waste”, “tunnel grouting materials”, “performance enhancement”, “process optimization”, “durability”, and “fluidity”. Various scientific databases, including “Web of Science”, “Google Scholar”, “Science Direct”, “Science Citation Index”, and “Scopus”, were utilized for the literature search. Based on this approach, a literature review of over 130 articles from the past decade was conducted.

This paper aims to provide a comprehensive overview of the recent studies on ISW grouting materials for tunnel engineering, analyze key factors influencing their properties, summarize the major issues and challenges, and discuss future research directions. It is divided into four main sections. Section 2 examines the feasibility of using different ISWs in tunnel grouting. Section 3 discusses the effects of ISW on key grout properties, including strength, fluidity, and durability. Section 4 identifies major challenges and suggests future work to realize the potential of ISW grouts. Section 5 provides the overall conclusions.

## 2. Feasibility of Industrial Solid Waste as Grouting Material

ISW primarily consists of waste slag, fly ash, and other ISW. The usage of ISW presents significant challenges due to its large output, varied types, and complex composition. A wide range of industrial wastes have been investigated for their viability as substitute materials in tunnel grouting formulations [53]. This section summarizes recent studies on the feasibility of incorporating different ISWs as partial replacements for cement or aggregates in grout mixtures. The effects of ISW composition and proportions on the properties of fresh and hardened grouts are also discussed.

### 2.1. Slag

**Figure 5:**
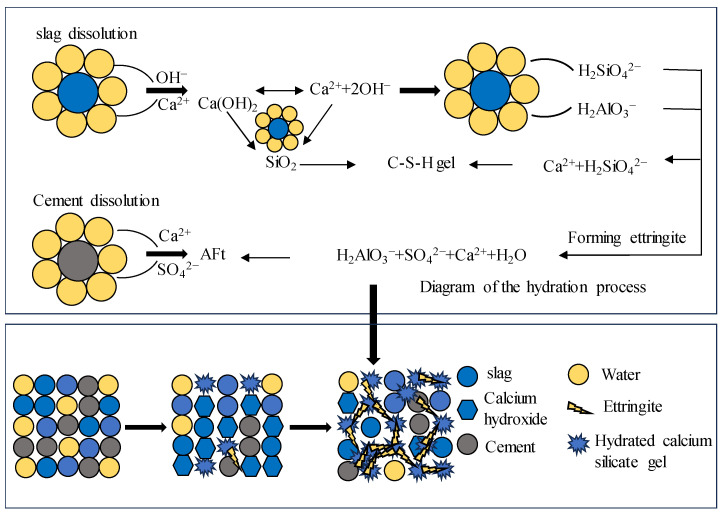
Schematic diagram of hydration reaction.

Slag is a by-product of iron and steel smelting processes. Research shows that slag powder can be used to partly replace cement in tunnel grouts. Lin et al. [54] found that slag undergoes hydration reactions when activated by cement and lime; the details are shown in Figure 5. The reaction products include calcium silicate hydrates (C-S-H) and calcium aluminate hydrates (C-A-H), which enhance strength. Li et al. [55] discovered that the compressive strength of the slag reached 20 MPa after the treatment, and the slag was suitable as a cementing material to replace a part of Portland cement. Prentice et al. [56] addressed the issues of a prolonged setting time and low early strength of (low clinker) slag cement by incorporating admixtures and implementing a compound preparation scheme. Through X-ray diffraction (XRD) testing and analysis, Yuyou, Zengdi, Xiangqian, and Haijun [40] explored the mechanism of tricalcium silicate in cement minerals to enhance the setting time and early compressive strength. Li et al. [57] optimized the particle size distribution of slag powder to achieve a 7-day grout strength exceeding 6 MPa with 30% slag incorporation.

Steel slag is a by-product of steel manufacturing. Its hydraulic properties allow it to be used in grouting. Ghouleh et al. [58] showed that hardened and graded steel slag aggregates improve grout stability. Up to 70% of natural aggregates can be substituted by steel slag. However, a higher steel slag content increases shrinkage. Li et al. [59] developed a cement-based composite material using ISW (steel slag) and sulphoaluminate cement clinker. This material leverages the cementitious activity of the silicon-aluminum component of steel slag, resulting in improved fluidity, stability, and resistance to ion erosion of the grouting material. André et al. [60] prepared grouts using ground granulated blast furnace slag and found that finely ground steel slag accelerates C-S-H gel formation, enabling early strength development.

**Figure 6:**
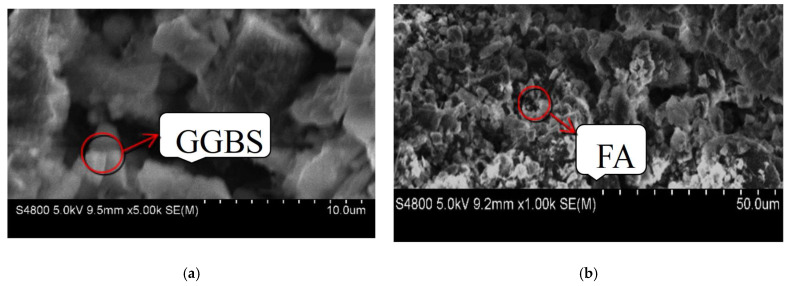
SEM images of solid precursors: (**a**) GGBS and (**b**) FA.

Gencel et al. [61] investigated the use of waste steel slag as a cementitious material in cement mortar. They analyzed the composition and structural characteristics of ISW and evaluated the active characteristics, curing degree, and performance of the activated cementitious body. Sun and Chen [62] studied the effects of steel slag fineness and the silicate modulus of sodium silicate solution on grout performance. A slag fineness of 7000 cm^2^/g and a silicate modulus of 2.5 were optimal. Yu et al. [63] suggested that a steel slag content between 30–50% in grouts provided favorable rheology, setting time, and strength. Grade S95 GGBS (ground granulated blast slag) and class F fly ash were used as solid precursors to produce the AAISW-based two-component grout in this study. GGBS particles are irregular and angular, while FA particles are spherical in different sizes, as shown in Figure 6.

### 2.2. Fly Ash

**Figure 7:**
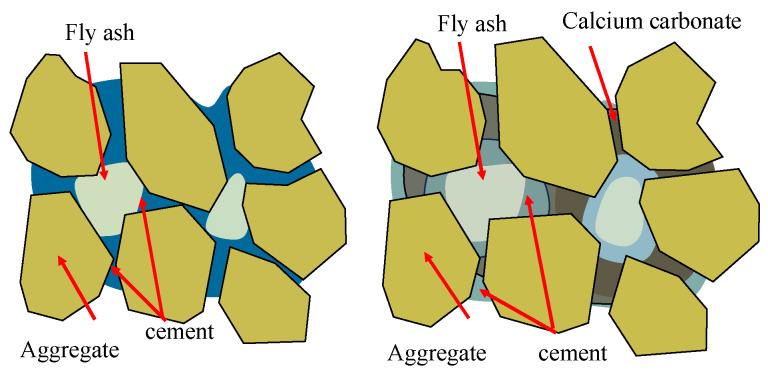
Schematic diagram of acidification reaction.

Fly ash is a fine powder produced from the combustion of coal. Fly ash exhibits strong pozzolanic reactivity and has been widely studied for grout applications. Pei et al. [64] found that class F fly ash can serve as a supplementary cementing material in grouting materials. The results revealed that incorporating 5% fly ash into the grouting material increased its compressive strength by more than 22% compared to the original grouting material. This enhancement is attributed to the filling of pores between aggregates by the calcium carbonate produced during the carbonation process of fly ash, facilitating the formation of a dense structure; the details are shown in Figure 7. Trimurtiningrum and Ekaputri [65] developed a grout mixture with a 70% class C fly ash replacement of cement. With a 10% gypsum addition, the grout achieved a 28-day strength of over 100 MPa. Li et al. [66] found that class C fly ash refinement is key to improving grout properties. Fly ash participation in pozzolanic reactions also enhances long-term strength. The optimal fly ash content is around 30% to 50% for strength and fluidity considerations.

### 2.3. Other ISWs

Apart from the aforementioned categories, other ISWs, including red mud, carbide slag, and water treatment residuals, have also been studied for grout production. Composite blends using two or more ISWs can further improve the grout properties compared to single admixtures. Huang et al. [67] investigated the influence of the slag content, the steel slag content and fineness on the autogenous shrinkage of Portland cement. The results indicated that the hydration activity of steel slag plays a crucial role in determining the autogenous shrinkage of the cement. Higher hydration activity leads to greater autogenous shrinkage. Cui et al. [68] employed grouting reinforcement technology and used sulphoaluminate cement as the primary material mixed with prepared talc-like substances. Deng and Zheng [69] utilized zinc slag as a raw material for grouting material derived from ISW. Zhang et al. [70] investigated the chemical structure, surface characteristics, and physical and mechanical properties of sulphoaluminate cement-based grouting materials. They enhanced the mechanical properties of these grouting materials by incorporating an appropriate amount of ISW admixture and water-soluble polyurethane.

Guo et al. [71] employed a significant amount of fly ash, coal gangue, a suitable quantity of desulfurization gypsum, and an alkali activator, along with a small amount of admixture, to prepare coal-based ISW grouting material. Additionally, they utilized response surface analysis to determine the optimal dosage for achieving the desirable fluidity, gel time, and other properties. Overall, an ISW content of around 40% is appropriate for balancing strength enhancement versus workability reduction. The effectiveness also depends on the ISW characteristics and activation methods.

Table 3 shows the feasibility results of different ISWs as grouting materials. It can be seen that using solid waste as grouting material can effectively reduce construction costs while facilitating the safe and efficient recycling of solid waste in engineering applications. Furthermore, treating ISW requires careful consideration of grouting performances in tunnel engineering, as well as the potential environmental pollution resulting from solid waste. The usage of ISW holds significant practical value and contributes to environmental protection, human health preservation, and sustainable development promotion.

## 3. Effect of Industrial Solid Waste on Performance of Grouting Material

With the rapid development of society, a generation of various industrial solid wastes (ISWs), including steel slag, slag, and fly ash, has surged. The large-scale stockpiling of these ISWs not only occupies land resources but also causes environmental pollution around the storage sites. Therefore, the comprehensive utilization of these resources is imperative. Currently, the incorporation of ISWs into building materials has become a research hotspot. Numerous studies have proven that the addition of appropriate amounts of ISWs to cement-based grouting materials can significantly reduce raw material costs while delivering enhanced mechanical performance [72]. This section examines how the incorporation of ISW influences the various grout properties essential for engineering performance, including strength, fluidity, impermeability, volume stability, and durability. The effects of the ISW type and dosage are analyzed based on recent studies.

### 3.1. Mechanical Strength of Grouting Material

Based on previous research, it summarizes the effects of different ISWs on the mechanical strength of cementitious grouting materials and discusses the mechanisms behind ISW incorporation to provide a theoretical basis for the resource utilization of ISWs.

#### 3.1.1. Industrial Solid Waste Incorporation and Process Optimization

The strength development in ISW grouts is linked to the pozzolanic reactivity of ISW components [73]. Si, Al, and Fe elements in ISW source materials can react with the Ca(OH)_2_ released during cement hydration to form additional strength-enhancing products. The porous structure of ISW particles also serves as nucleation sites, refining pore structure and increasing density.

Common ISWs, such as slag, fly ash, and steel slag, are widely used in cement-based grouting materials. For instance, the combination of slag powder and cement can produce materials with high early strength for full tailings filling, while the mixture of steel slag slurry and cement exhibits significantly improved compressive strength after carbonation [74]. The proportional incorporation of Bayer red mud, carbide slag, and desulfurized gypsum continuously enhances material strength during the carbonation process due to the gradual formation of calcium carbonate. At the same time, the Bayer process will generate the following types of industrial waste (including arsenic waste, silica ferrous slag, slurry wastewater, etc.), and to minimize the environmental impact and waste of resources, the relevant industrial waste needs to be treated and managed appropriately.

Cao et al. [75] combined slag powder and P.O. 42.5 cement to develop a cementitious material with specific early strength for full tailings filling. The composition consisted of 60% slag, 15% cement, 10% hemihydrate gypsum and desulfurization gypsum, and 5% lime. Xue et al. [76] utilized slag powder, along with clinker and gypsum, to develop a cementitious material for the purpose of whole tailings filling. This material exhibited a remarkable strength that was 2.7 times higher than that of cement-based filler in similar conditions. Furthermore, it proved to be a cost-effective alternative, with a significantly lower cost ranging from 40% to 45% of traditional cement. Sha et al. [77] combined pure steel slag slurry and Portland cement to form a steel slag mixture. After carbonization, the compressive strength of the mixture significantly improved. Deng et al. [78] developed a graded tailings filling cemented body using lead-zinc smelting slag, clinker, sodium silicate, gypsum, and other activating materials, with a cement–sand ratio of 1:6. The strength of the cured samples after 3 days reached 2.68 MPa, while after 28 days, it reached 3.97 MPa, representing a significant 21% increase compared to traditional P.O. 42.5 cement. Li et al. [79] developed a dual-liquid grouting material, incorporating industrial waste residues such as steel slag, slag, fly ash, and water glass. The material exhibited a gel time of 3–300 s and a strength of 15–25 MPa. This material significantly enhances the utilization of industrial waste residues and reduces cement grouting consumption. Lahalle et al. [80] developed an alkali-activated polymer double-liquid grouting material using sodium silicate-activated slag and metakaolin. They proposed that when the mass ratio of slag to metakaolin was 1:2, and the volume content of glass was 20%, the compressive strength at 3 days and 28 days reached 7.23 MPa and 17.26 MPa, respectively. Bai et al. [81] prepared a fly ash-based grouting material with an initial setting time of 13 min, a final setting time of 20 min, and a compressive strength of 104.5 MPa. Wan et al. [82] addressed the slow hydration rate of a steel slag–slag–barium slag composite grouting material by utilizing desulfurization gypsum as an activator. The results showed a 21% increase in the 28-day strength. Sha et al. [83] prepared a new type of cement-fly ash-modified sodium silicate grouting material. The results showed that the initial setting time increased, and the final setting time decreased with an increase in the volume fraction of modified sodium silicate. The gel time was proportional to the mass fraction of fly ash in the powder, while the slurry’s fluidity was proportional to the volume fraction of modified sodium silicate and the mass fraction of fly ash in the powder.

Further enhancement of grouting material performance can be achieved by optimizing the addition amounts, combinational types, and process parameters of ISWs. Zhu et al. [84] prepared cementitious materials using slag and fly ash as raw materials under alkali activation. The findings revealed that the compressive strength initially increased and then decreased with a decreasing fly ash content when 3% sodium hydroxide (by mass) was used as an alkali activator and the water-to-solid ratio was 0.4. Juan-hong et al. [85] prepared a grouting material by mixing red mud, carbide slag, and desulfurized gypsum at a mass ratio of 74:11:15. The results demonstrated that the content of calcium carbonate in the material increased with carbonization time, leading to a gradual increase in compressive strength. Zhang et al. [86] developed a novel double-liquid grouting material with an adjustable gel time, high consolidation strength, and a stone rate of up to 100%. This material was produced by combining industrial waste residue with Portland cement clinker, optimizing particle size distribution, and alkaline-activating cementitious activity.

Carvalho et al. [87] investigated the impact of steel slag replacement on the performance of composite filling cementitious materials. The findings revealed a pattern of initial shrinkage followed by expansion as the steel slag replacement amount increased, with shrinkage reaching its lowest point at 83%. Chao et al. [88] conducted a comprehensive tailings filling test using a cost-effective grouting material they developed, primarily composed of fly ash, slag powder, carbide slag, and a composite activator. The results demonstrated that with a cementing agent-to-tailings ratio of 1:4–1:8 and a slurry concentration of 67–70%, the 28-day compressive strength ranged from 0.68–2.63 MPa. Ehsani et al. [89] examined the water absorption, fluidity, and compressive strength of a novel fly ash-cement grouting material and determined that optimal performance was achieved with a fly ash ratio of 17–20% and a water–cement ratio of 0.65–0.7. Rehman et al. [90] examined the performance of a steel slag-modified silicate-sodium silicate double-liquid grouting composite material. Their research determined that the optimal water–binder ratio ranged from 0.6–0.8, the ideal volume ratio of sodium silicate to the slurry was 1:4–1:6, and the steel slag content constituted 50–80% of the cement mass. After 3 days of curing, the material with the ratios achieved an early strength exceeding 40 MPa. To enhance the comprehensive utilization of solid waste, Xue et al. [91] conducted an optimization test on subsection iron removal from steel slag and investigated the influence of steel slag powder on the compressive strength of the grouting material. The findings revealed that through sectional magnetic separation, high-performance steel slag powder with less than a 0.5% metal iron content could be obtained. Additionally, when the specific surface area of the steel slag powder was 1:2.5, the material achieved improved 3-day and 28-day strength simultaneously.

Gopinathan and Anand [92] investigated the mechanical properties of grouting materials with a high content of slag powder. The results demonstrated that as the slag incorporation rate increased, the slurry density gradually increased, porosity decreased, and strength increased. Additionally, under low-temperature conditions, the high-content slag powder grouting material exhibited an improvement in compressive strength by 18–27% compared to conventional grouting materials.

Based on a comprehensive analysis of steel slag powder characteristics, Liu and Guo [93] incorporated it into a steel slag-cement and steel slag-mineral powder-fly ash composite system. The research indicated that the optimal replacement rate of steel slag powder in cement was 15%, and the optimum ratio of steel slag powder to mineral powder-fly ash was 1:2:1. Furthermore, the use of Na_2_SO_4_ as an activator in the steel slag powder-cement composite system effectively improved the early strength of cementitious materials.

#### 3.1.2. Mechanisms of Performance Enhancement

The incorporation of ISWs promotes cement hydration reactions and generates more hydration products, such as ettringite and C-S-H gel. For example, the addition of steel slag and slag induces hydration reactions when activated by cement [94]. The initial hydration of steel slag promotes the depolymerization of steel slag, which subsequently reacts with cement to generate ettringite network structures that are subsequently filled by C-S-H gel, thereby increasing the density of the hardened paste system.

Li et al. [95], guided by grouting engineering performance, primarily utilizes red mud from ISW as the main raw material to prepare a red mud-based grouting material in collaboration with various solid waste materials. The occurrence of free water, the hydration exothermic law, hydration kinetics characteristics, alkaline components, and the heavy metal content of the slurry are analyzed. The hydration mechanics characteristics of the red mud-based grouting material are determined, and its hydration mechanism is revealed. The hydration dynamics characteristics are analyzed using the Krstulovic–Dabic model.

Zhang et al. [96] found that different reaction degrees and sizes of embedded microstructures were observed in fly ash particles at different ages, adversely affecting the mechanical properties of the materials. The activation degree of fly ash gradually increased with age, contributing continuously to the strength development in the later stages. This is due to the fact that with the increase of age, the active substances in the fly ash (the activated substances refer to the components in the fly ash that can react with water to generate cementitious substances, which play an important role in promoting the development of the later strength of the material) will be gradually depleted, and the degree of activation will be gradually reduced, which will adversely affect the development of the later strength.

Liu et al. [97] investigated the hydration reaction process of a clinker-free cementitious system comprising steel slag, slag, and gypsum. The results revealed that an increase in the specific surface area of steel slag powder enhanced the compressive strength of non-clinker concrete but reduced its slump. The network structure formed by ettringite during the early stages played a vital role in the material’s early compressive strength, while the hydration products and C-S-H gel continuously filled the gaps in the hardened paste, enhancing the overall compressive strength of the material.

From the above studies, it is easy to see that the use of ISW as grouting material can not only realize the comprehensive utilization of resources but also have significant economic benefits. However, although the existing studies on various ISW-modification mechanisms can provide some theoretical references for practical projects, the systematic selection of ISW materials and optimal dosage to produce low-cost and high-performance grouting materials requires further exploration.

### 3.2. Fluidity of Grouting Material

Grouting applications in tunnels, foundations, and structural repairs demand controllable fluidity and strength [98]. High fluidity is required during grout injection to achieve good penetration and filling capacity. ISW particles can improve particle packing and lubrication between cement grains, enhancing grout fluidity [99]. However, an excessive ISW dosage can sharply increase viscosity due to a higher specific surface. This section comprehensively examines the interconnected factors influencing the fluidity of grouting material.

The effects of alkali metal cations (K^+^, Na^+^) on slurry rheology and setting behaviors were evaluated. Li et al. [100] investigated the slurry viscosity and setting time of grouting materials mixed with different alkali metal cations using Fourier transform infrared spectroscopy (FTIR) and viscosity tests. The results indicate that when the ratio of alkali metal cation (K^+^) to silicon ion is less than 1.5, the initial setting time of alkali slag grouting material can be effectively prolonged. Additionally, the addition of an appropriate amount of K^+^ can effectively reduce the viscosity of the solution and improve the material’s fluidity.

The alkali type/content regulated the viscosity and setting, enhancing workability below a threshold. Fly ash and slag modulated the workability and accelerated the strength gain in optimal dosages. Moghadam et al. [101] activated coal gangue, steel slag, and fly ash using NaOH and Na_2_SO_4_ to prepare cement-free, controllable, low-strength grouting material. When the mass fraction of fly ash is below 40%, the workability (fluidity) of the grouting material meets the ACI (American Concrete Institute) specifications. The phase composition of reaction products in the routing material system is influenced by the mass ratio of steel slag to fly ash. Xu et al. [102] studied the influence of fly ash on the working performance of grouting materials. The results demonstrate that the addition of fly ash significantly improves the fluidity of the materials, and the fluidity increases gradually with the increasing fly ash content up to 40%, resulting in good working performance. ElKhatib et al. [103] successfully prepared various compaction grouting materials for tunnel grouting by utilizing steel slag, fly ash, and other raw materials based on the activity characteristics of steel slag. The results show that the addition of steel slag improves the fluidity of the grouting material, reduces shrinkage, accelerates setting time, and significantly enhances the 7-day and 28-day strength.

Xiang et al. [104] investigated the effect of large amounts of mineral admixtures, such as fly ash and mineral powder, on the performance of grouting materials. The results indicate that a material with a composition of 30% fly ash and 10% mineral powder achieves optimal fluidity. Wu et al. [105] conducted an experimental study on the basic performance of solid waste grouting filling material using rubber particles, fly ash, and clay as the main materials. The results show that a grouting slurry with a fluidity of 293 mm, a 28-day compressive strength of 11.7 MPa, and an impermeability pressure of 0.8 MPa can be achieved when the content of rubber particles is 20%, fly ash is 65%, and clay is 15%.

In summary, the above studies have elucidated the rheological properties, microstructure-property linkages, and composition-property linkages of ISW grouting materials, providing theoretical guidance for the design of sustainable materials. However, due to the limitations of microscopic equipment and numerical simulation computational capabilities, the mechanistic explanations of the above phenomena have not yet gained a unified perception.

### 3.3. Durability of Grouting Material

Grouting materials are widely used in tunnel engineering to consolidate surrounding rock and soil, control groundwater infiltration, and prevent corrosion. However, grouting materials often face harsh and complex service environments that can impact their durability and performance. This section categorizes and summarizes the recent studies on various factors influencing the durability of grouting materials, including volume stability, water corrosion resistance, impermeability, sulfate attack resistance, and freeze–thaw resistance.

#### 3.3.1. Volume Stability

Volume stability is critical for grouting materials to avoid excessive shrinkage and cracking. Given that the service environment of grouting material is dynamic and intricate, the volume stability of the grout body is closely associated with the engineering’s service life. Excessive shrinkage of the grout can lead to crack formation, which in turn can cause leakage and intensify the erosion caused by corrosive ions. He et al. [106] found that high early strength cement grouts exhibit micro-expansion without compromising strength. This expansibility tends to stabilize within the initial 7 days without compromising the subsequent strength of the grout body. Furthermore, micro-expansion also contributes to improving the compactness of hardened slurry. Li and Hao [107] showed that the early strength agent increased the early chemical shrinkage of the double-liquid sulphoaluminate grouting material. Under standard oxidation conditions, the grout experienced volume expansion; however, under dry air curing, it exhibited volume shrinkage. Borçato et al. [108] determined that the shrinkage rate decreases with increasing metakaolin content in geopolymer grouts. The results demonstrated that the shrinkage rate of the grout body decreased with increasing high terrestrial content. However, when the content exceeded 30%, the effect began to weaken. The application of industrial solid waste flue gas desulfurization gypsum (FGDG) in magnesium oxy-sulfate cement (MOSC) was investigated by Li et al. [109]. The results showed that the volume stability of the mixture with the optimum admixture was better at the curing temperature of 40 °C, and its 28-day compressive strength could reach 56.6 MPa, which was 12.5% higher than that of the curing condition at 20 °C, and the softening coefficient could reach 0.99. Meanwhile, it was also confirmed that more hydration products (5Mg(OH)_2_·MgSO_4_·7H_2_O) were produced in the mixture under the curing condition of 40 °C through SEM and XRD, and its structure is denser.

#### 3.3.2. Water Corrosion Resistance

Groundwater can cause the gel product in the hardened grout stone body to decompose and dissolve through physical and chemical actions, resulting in a decrease in the mechanical properties of the stone body [110]. This phenomenon is known as water corrosion. Therefore, flowing water can cause dissolution and deterioration of grout gel products and strength loss. Bentz and Garboczi [111] identified calcium hydroxide as vulnerable to leaching and dissolution under the influence of flowing water. This is especially true under conditions of high permeability and high-water pressure in hardened cement paste, where water can infiltrate the stone body, dissolve calcium hydroxide, and then permeate out, leading to a decrease in the compactness and strength of the hardened paste. Furthermore, the dissolution of calcium hydroxide leads to a decrease in the alkalinity of the matrix, which can further cause the decomposition of other hydration products.

Several comparative studies evaluated the water corrosion resistance of various grouts using strength loss, ion leaching, and microscopic analysis. Sun et al. [112] have analyzed the water corrosion resistance of different types of grouting materials, including single-fluid cement slurry, cement-water glass slurry, and industrial waste residue-based slurry. Zhao et al. [113] evaluated the water corrosion resistance characteristics of clay-cement paste slurry, clay-cement stabilized slurry, and single-fluid cement slurry by measuring the amount of calcium oxide dissolved in immersion water; they evaluated the water corrosion resistance characteristics of these three types of slurries. It was found that the viscosity of cement paste slurry exhibited the best water corrosion resistance. Weldes and Lange [114] conducted experimental research and evaluation using indexes such as the strength loss rate, sodium ion solidification rate, silicate dissolution amount, electrical conductivity, and total mass of dissolved matter. They compared these results with those of cement-based grout and discussed the reasons for the improved water corrosion resistance of industrial waste residue-based grout through microscopic tests. Gebauer and Coughlin [115] performed water corrosion resistance tests on hardened samples of different types of grouts, including pure cement grout, cement-water glass grout, cement mortar, and a newly developed grout. The above results suggested that industrial waste-based grouts and viscosity-modified grouts exhibited improved resistance.

#### 3.3.3. Impermeability

Impermeability is an important performance index of the hardened stone body of grouting materials. It reflects how difficult it is for liquids or ions to infiltrate, diffuse, or migrate within the hardened stone body under pressure, chemical potential, or electric field. A grout with good impermeability usually has a relatively dense microstructure, making it resistant to penetration by external liquids and corrosive ions, which can lead to the deterioration of grouting material performance. Current studies analyzed the effects of density, mineral admixtures, mix proportions, and additives on the impermeability of specialized grout mixes, identifying the optimal parameters. Wang et al. [116] conducted research on the relationship between impermeability and density of ISW grouting materials. The results indicate that the density of polymer materials (non-aqueous reactive polymer grouting materials represented by polyurethane foam) is generally in the range of 0.1–0.3 g/cm^3^, and the initial seepage pressure is in the range of 0.3–0.7 MPa, which can bear 30 to 70 m’s head pressure when seepage grouting is carried out in hydraulic engineering. In practical applications, the amount of polymer grouting should be controlled, and the density of the polymer material should be adjusted according to the actual needs of different projects to meet the needs of seepage control in different projects. Mangat and Khatib [117] studied the influence of fly ash, silica fume, and slag powder on the impermeability of cement paste using a mortar permeameter. Their findings indicated that each material had different optimal replacement rates, which could optimize the impermeability of the hardened slurry. Shana and Jeffrey [118] analyzed the influencing factors of the permeability coefficient of slag cement slurry. They concluded that slag cement slurry prepared under suitable ratio conditions exhibited good impermeability and had broad application prospects. Zhang et al. [119] highlighted that alkali-activated cementitious materials prepared from dolomite, sodium carbonate, and calcined bentonite possess excellent water resistance. Zhao et al. [120] used the softening coefficient as a parameter to characterize the water resistance of the materials. The study found that incorporating 10–20% rice husk ash improved the water resistance of the prepared cementitious materials significantly. Yang et al. [121] discovered that after undergoing the same high-temperature test, the damage in terms of compressive strength and bond strength was lower in alkali-activated mortar compared to cement mortar, indicating better high-temperature resistance. Li et al. [122] investigated the influence of the water-binder ratio and the ratio of liquid A to liquid B on the impermeability of hardened slurry samples during the preparation of cement-sodium silicate double slurry. The maximum impermeability pressure of the prepared samples reached 1.6 MPa. Porbaha et al. [123] developed a testing device for measuring the permeability coefficient of hardened grout to study the impermeability of clay-solidified grout. Song et al. [25] used a new additive, called Z201, to prepare a grouting material with excellent impermeability for shield tunnel walls. When the content of additive Z201 was 10%, the impermeability pressure reached 0.8 MPa.

#### 3.3.4. Sulfate Attack Resistance

Groundwater sulfates can react with grout hydration products, causing expansion, strength loss, and cracks [124]. Groundwater used in grouting often contains sulfate substances such as sodium sulfate and magnesium sulfate. When the content of these sulfate substances is high, it can lead to the erosion and deterioration of the hardened paste over time, resulting in a decrease in its strength. Sulfate erosion can be categorized into internal erosion and external erosion based on the source of sulfate ions [125]. Internal erosion occurs when sulfate ions are present within the hardened paste itself, while external erosion is caused by sulfate ions from the surrounding environment. The process of external sulfate erosion can be divided into two stages: infiltration of sulfate ions into the matrix of the hardened paste from groundwater, which is influenced by the impermeability of the hardened paste, and the reaction between sulfate ions and substances in the hardened paste matrix. The corrosion mechanism of sulfate on cement-sodium silicate grout was studied [32]. It was found that the gel products of traditional cement-based grout, mainly Ca(OH)_2_ and C-S-H with a high Ca/Si ratio, react with sulfate ions to form expansive crystals, leading to the destruction of the matrix structure. Additionally, the stability of the C-S-H gel with a high Ca/Si ratio is poor, and the reaction consumption of Ca(OH)_2_ reduces the alkalinity in the matrix, causing decomposition and destruction of the C-S-H gel. Carde et al. [126] identified calcium hydroxide and C-S-H as vulnerable and described degradation mechanisms. Yoshikawa and Toda [127] suggested that low concentrations of sulfate solutions can promote the early-stage strength development of hardened paste, while high concentrations of sulfate solutions can cause a decline in strength. Kourounis et al. [128] found that incorporating high-viscosity materials and steel slag into traditional cement-sodium silicate grout as partial replacements for cement can enhance the sulfate corrosion resistance of the grout. Therefore, it is important to conduct the appropriate research to develop grouting materials with improved sulfate corrosion resistance.

#### 3.3.5. Freeze–Thaw Resistance

Karakurt and Bayazıt [129] compared the freeze–thaw resistance of alkali-activated concrete (with fly ash partially replacing kaolin) with ordinary Portland cement concrete. They found that ordinary Portland cement concrete reached a mass loss rate of 5% after 45 freeze–thaw cycles, while alkali-activated concrete required 98 freeze–thaw cycles to reach the same mass loss rate. Liu et al. [130] studied the effect of silica fume on the freeze–thaw resistance of alkali-activated fly ash/slag cementitious materials through experiments. The results showed that the addition of silica fume led to more damage and deterioration of the hardened samples under freeze–thaw cycles.

Aiken et al. [131] investigated the effects of freeze–thaw cycles on the mechanical properties of a grouting material composed of basalt fiber, slag powder, and fly ash. The results indicated that the material with a basalt fiber content of 0.18 vol% exhibited superior frost resistance, as well as the highest compressive and tensile strengths compared to other fiber contents under the same freeze–thaw cycles.

Hou et al. [132] emphasized that increasing the slag content in alkali-activated fly ash/slag cementitious materials can reduce the porosity and pore diameter of the hardened sample matrix, thereby enhancing the freeze–thaw resistance of the materials.

Vegas et al. [133] conducted 300 freeze–thaw cycle tests on alkali-activated fly ash mortar and traditional Portland cement-based mortar. It was concluded that alkali-activated materials exhibit better freeze–thaw resistance than traditional Portland cement.

Slavik et al. [134] investigated the freeze–thaw resistance of alkali-activated fluidized bed combustion bottom ash cementitious material. The results showed that the compressive strength of hardened samples retained over 80%, even after 50 freeze–thaw cycles, indicating good freeze–thaw resistance. These studies collectively suggest that alkali-activated ISW cement materials generally exhibit superior freeze–thaw resistance compared to ordinary Portland cement-based materials. The slag content, silica fume addition, and other compositions affect the performance of the grouting materials.

#### 3.3.6. Corrosion Resistance

Several research studies suggested that alkali-activated materials, after hardening, exhibit high mechanical strength and corrosion resistance compared to traditional cement-based materials. Wu et al. [135] studied the high-temperature resistance of alkali-activated fly ash/slag-based cementitious materials. They noted that the critical temperature for the degradation of physical and mechanical properties of the alkali-activated materials prepared from fly ash/slag is between 400–600 °C. Kanaan [136] compared the sulfate attack resistance of alkali-activated high territory/slag-based paste materials with pure cement-based paste materials. The results showed that the paste samples containing slag exhibited the best sulfate attack resistance, followed by pure kaolin samples, while the cement-based samples had the least resistance to high temperatures. These findings suggest that alkali-activated materials, represented by ISW-based grouting materials, offer improved performance in terms of sulfate attack resistance, making them advantageous for various applications in construction and engineering. Fernández-Jiménez et al. [137] investigated the corrosion resistance of alkali-activated cementitious materials compared to cement-based cementitious materials. The results demonstrated that the corrosion resistance coefficient of alkali-activated cementitious materials was significantly higher than that of cement-based materials. Meanwhile, Xin et al. [138] found that under corrosive conditions, the appearance damage of cement-based samples was more severe compared to alkali-activated fly ash-based cementitious material samples. This can be attributed to the gel products in cement-based samples, primarily composed of Ca(OH)_2_, C-S-H gel, and C-A-H gel, with a high Ca/Si ratio. These gels are stable in alkaline environments but easily decompose and deteriorate in acidic environments. These findings suggest that alkali-activated cementitious materials possess superior corrosion resistance compared to cement-based materials, making them more suitable for applications where exposure to corrosive environments is a concern. From the above studies, it is easy to see that ISW grouting materials possess superior durability properties under specific conditions compared to ordinary materials. However, conventional modification methods can only improve a certain property, and it is necessary to find a convenient and efficient method to improve the overall durability of ISW grouting materials.

## 4. Challenges and Future Prospects

The rapid expansion of infrastructure has resulted in a global increase in tunnel construction. Concurrently, significant industrialization has generated substantial amounts of industrial solid waste (ISW), leading to resource wastage and environmental pollution. Utilizing ISW as grouting materials in tunnels offers an eco-friendly and cost-effective solution to address these interconnected issues. However, several key challenges must be addressed before the widespread application of ISW grouting materials can be achieved.

One primary challenge is ensuring a consistent high performance of ISW grouting materials. Due to the variability in ISW composition and properties, the mechanical strength, fluidity, impermeability, durability, and other engineering properties of ISW grouts can be unstable. Thorough characterization and optimal mix design are necessary to meet the demanding performance criteria. Research should systematically investigate the complex effects of different ISW types, proportions, chemical activators, and preparation processes on grout properties.

Another major concern is the environmental impact and ecological safety associated with the use of ISW grouting materials. While intended to mitigate pollution, the improper use of ISW with high levels of heavy metals or other hazardous substances can introduce new environmental risks. Comprehensive assessment frameworks must be established to evaluate the leaching potential and ecological effects of ISW grouts. Standardized procedures are required to treat ISW and purify grout mixes to comply with health and safety regulations. Further research should focus on developing high-quality grouting materials from industrial waste.

Furthermore, there is currently a lack of specific international codes, standards, and guidelines regarding ISW grout production and quality control. Extensive mechanical and durability testing needs to be conducted to determine reasonable technical specifications for engineering properties, mix proportions, construction methods, and acceptance criteria. Systematic field testing and monitoring in pilot projects are essential to validate ISW grout performance. The accumulated experience should be formulated into detailed protocols and regulations to ensure quality assurance and safe, widespread implementation.

Advanced microstructure analysis can provide valuable insights into the composition–structure–property relationships of ISW grouting materials, guiding the optimization of their engineering properties. Some key ways in which advanced microstructure analysis aids in this optimization include:

(1) Scanning electron microscopy (SEM) coupled with energy-dispersive X-ray spectroscopy (EDS) can characterize the elemental distribution and chemical composition of different phases and reaction products in the grout’s microstructure, revealing the role of specific ISW particles and hydration products in enhancing properties.

(2) Transmission electron microscopy (TEM) enables nanoscale examination of C-S-H gels and other nanoscale hydrates, facilitating an understanding of macro-scale strength and impermeability.

(3) Mercury intrusion porosimetry quantifies the pore size distribution, allowing for the study of pore parameters like total porosity, critical pore diameter, shape, and connectivity, which helps establish correlations between permeability and durability.

(4) XRD identifies crystalline reaction products, providing insights into strength development mechanisms by tracking formations such as calcium silicate hydrates and calcium aluminate hydrates at different curing ages.

(5) Fourier transform infrared spectroscopy detects chemical bonding information and can monitor the progress of pozzolanic reactions and hydration processes over time.

(6) Nuclear magnetic resonance spectroscopy analyzes silicate polymerization, revealing the cross-linking density of the C-S-H gel phase, which largely controls strength.

(7) Mass spectroscopy accurately identifies the ion types and concentrations leached from grout samples in durability testing, aiding in the evaluation of resistance mechanisms.

By correlating advanced microstructural observations with measured engineering properties, the optimal type and dosage of ISW can be determined to tailor grout behavior for specific applications. The micro–macro linkages provided by sophisticated analysis are invaluable for designing high-performance and durable ISW grouting materials.

Advanced techniques also offer opportunities to enhance ISW grouts. For example:

(1) Optimizing the particle size distribution through fine grinding and classification can improve particle packing density, rheology, and strength. Meanwhile, novel admixtures and nano-additives may regulate the setting time, reduce shrinkage, and enhance impermeability.

(2) Advanced sensors and monitoring technologies are utilized to monitor the rheological properties, uniformity, and curing process of the grouting process in real time for precise control and adjustment.

(3) Using 3D-printing technology, the geometry and microstructure of grouting materials can be precisely controlled to improve the mechanical properties of grouting and process efficiency.

(4) Microencapsulation technology encapsulates additives in tiny capsules, allowing for a precise controlled release effect that improves the performance and stability of the grouting material.

(5) Using computational simulation and numerical analysis methods, the grouting process can be accurately modeled and simulated to better understand material behavior and optimize the grouting design.

In the future, the integration of diverse industrial wastes into optimal grout design will be facilitated by advanced technical means, promoting sustainable infrastructure construction. More research should be directed towards the development of multifunctional grouts with self-sensing, crack-healing, and microbial properties for the creation of “smart tunnels”. Resource utilization can be maximized through the recycling and reusing of excavated soil, demolition waste, and industrial waste. With persistent research efforts and interdisciplinary collaboration, the evolution of ISW grouting technology will benefit both the industry and the environment.

## 5. Conclusions

This comprehensive review of recent studies on utilizing industrial solid waste (ISW) in tunnel grouting materials demonstrates the promising potential of this sustainable approach. However, further research and development are needed to address the existing limitations and knowledge gaps. The main findings are as follows:

(1) The existing literature is well established on a variety of ISWs (including slag, fly ash, steel slag, red mud, silica fume, calcium carbide slag, etc.) as partial substitutes for cement in grout formulations. At the same time, depending on the ISW reactivity, ISW can be mixed up to 50%. Composite mineral admixtures can participate in the hydration reaction to produce strength-enhancing gel products, such as C-S-H and C-A-H. Therefore, although there is no uniform understanding of ISW as a grouting material, there is no doubt that the use of the right type of ISW in the right dosage can reduce the cost of grouting and provide an environmentally friendly recycling solution for the large quantities of ISW that are generated globally.

(2) The mechanical properties, fluidity, volume stability, impermeability, and durability of ISW grouts depend on the specific type and dosage of ISW. The development of strength relies on the pozzolanic reactivity of the ISW components. To achieve a balance between strength and workability loss, an optimal range of industrial solid waste (ISW) content is considered to be 20–50%. The particle size distribution and shape of ISW particles affect the rheology of the grout. It is crucial to optimize the mix design, considering the characteristics of ISW to meet the engineering requirements. Microstructure analysis reveals that ISW hydration and refinements in the micro-scale pore structure contribute to enhanced macro-scale properties.

(3) Before an extensive application of ISW grouts can take place, several technological barriers need to be addressed. The variability and uncertainty of ISW properties make it challenging to achieve consistent grout quality. There is a lack of standardized codes, specifications, and quality control procedures in this area. It is necessary to conduct rigorous assessments to evaluate the ecological impacts. The use of advanced computer simulation and 3D-printing techniques might enable the precise control of properties customized to local conditions.

In summary, the use of ISW as substitute raw materials in tunnel grouting offers significant potential for resource recycling and pollution mitigation. With ongoing research to address the current challenges, ISW grouting technology can be continually enhanced to deliver sustainable and high-performance solutions for underground construction globally. This review provides valuable insights to support further exploration in this emerging field.

## Figures and Tables

**Table 1:** The summary of case studies on the application of grouting materials.

Application Area	Engineering Environment	Material Composition	Reasons For Selection	Ref.
Changsha, Hunan Province, China	Karst ground	Sodium bentonite, lignin, meta-aluminate, cement	Better stability	[31]
Qingdao, Shandong Province, China	Marine environment	OPC, sodium silicate	Higher erosion resistance and better strength	[32]
Changsha, Hunan Province, China	Loose ground	Cement, clay, sodium bentonite, gypsum	Better stability and permeability	[33]
Wuhan, Hubei Province, China	High-pressure, water-rich strata	OPC, sand, fly ash, silica fume, steel slag, sodium bentonite	Better stability	[34]
Wuhan, Hubei Province, China	Under river	OPC, steel slag, fly ash, slag, metakaolin, sodium silicate,	Better corrosion resistance	[35]
Nanjing, Jiangsu Province, China	Soft soil strata	OPC, fly ash, hydrated lime, sand, clay, sodium bentonite,	Good workability and corrosion resistance	[36]
Wuhan, Hubei Province, China	Water-rich strata	OPC, sand, fly ash, silica fume, SPs, HEC	Good flow and strength properties	[37]
Xi’an, Shaanxi Province, China	Loess ground	OPC, sand, fly ash, sodium bentonite, micro-expansive agents	Higher strength and good workability	[38]
Beijing, China	Water-rich strata	OPC, fly ash, slag, sodium silicate, sodium bentonite, retarder agents	Better liquidity performance	[39]
Beijing, China	Sulfate-rich environment	Cement, fly ash, slag, sodium silicate, sodium bentonite	Good flow and strength properties	[40]
Chengdu, Sichuan Province, China	Complex ground	OPC, CSA, silica fume, early strength agent	High early strength and better durability	[41]

**Table 2:** Specific chemical composition of solid waste.

Species	Fe_2_O_3_ /%	FeO /%	Al_2_O_3_ /%	CaO /%	MgO/%	SiO_2_ /%	MnO /%
Cyclone ash	66.45	10.42	3.21	1.74	0.32	4.91	0.12
Sintered dust	64.89	2.8	2.9	14.2	3.05	6.35	0.23
Converter Dephosphrized steel slag	3.83	12.52	3.55	39.66	3.02	15.25	6.08
Species	TiO_2_ /%	Na_2_O /%	K_2_O /%	ZnO /%	Cl /%	P_2_O_5_ /%	Other /%
Cyclone ash	0.08	0.16	0.15	0.31	0.59	0.05	11.49
Sintered dust	0.09	0.06	0.3	0.03	0.28	0.06	4.76
Converter Dephosphrized steel slag	2.03	0.3	0.36	0.014	-	6.58	6.8

**Table 3:** Feasibility summary of various ISW materials as grouting materials.

ISW Type	Procedure	Improved Performance	Feasibility
Slag	Composite addition with admixture	Enhanced strength performance	Feasible
Steel slag	Optimize particle size distribution	Fluidity, stability, and resistance to ion erosion	Feasible
Fly ash	The amount of incorporation in the appropriate range	Increase compressive strength	Feasible
Other ISWs (including red mud, carbide slag, water treatment residuals, etc.)	Two or more than two types of composite incorporation	Improve fluidity, gelation time, and other working properties.	Feasible

## Data Availability

Not applicable.
